# Prospecting for candidate molecules from *Conus virgo* toxins to develop new biopharmaceuticals

**DOI:** 10.1590/1678-9199-JVATITD-2022-0028

**Published:** 2022-12-16

**Authors:** Anas A. Mohamed, Zohour I. Nabil, Mohamed S. El-Naggar

**Affiliations:** 1 Zoology Department, Faculty of Science, Suez Canal University, Ismailia, Egypt.; 2 Pharmacognosy Department, Faculty of Pharmacy, The British University in Egypt, Cairo, Egypt.

**Keywords:** Conus virgo, Pharmacological activity, Analgesic action, Antipyretic agent, Anti-inflammatory effects, Oxidative stress

## Abstract

**Background:**

A combination of pharmacological and biomedical assays was applied in this study to examine the bioactivity of *Conus virgo* crude venom in order to determine the potential pharmacological benefit of this venom, and its *in vivo* mechanism of action.

**Methods:**

Two doses (1/5 and 1/10 of LC_50_, 9.14 and 4.57 mg/kg) of the venom were used in pharmacological assays (central and peripheral analgesic, anti-inflammatory and antipyretic), while 1/2 of LC_50_ (22.85 mg/kg) was used in cytotoxic assays on experimental animals at different time intervals, and then compared with control and reference drug groups.

**Results:**

The tail immersion time was significantly increased in venom-treated mice compared with the control group. Also, a significant reduction in writhing movement was recorded after injection of both venom doses compared with the control group. In addition, only the high venom concentration has a mild anti-inflammatory effect at the late inflammation stage. The induced pyrexia was also decreased significantly after treatment with both venom doses. On the other hand, significant increases were observed in lipid peroxidation (after 4 hours) and reduced glutathione contents and glutathione peroxidase activity, while contents of lipid peroxidation and nitric oxide (after 24 hours) and catalase activity were depleted significantly after venom administration.

**Conclusion:**

These results indicated that the crude venom of *Conus virgo* probably contain bioactive components that have pharmacological activities with low cytotoxic effects. Therefore, it may comprise a potential lead compound for the development of drugs that would control pain and pyrexia.

## Background

To meet the progressive global demand for more sensitive and potent drugs capable of controlling pain and inflammation, despite progressive ineffectiveness of recent synthetic drugs, natural products are currently expected to offer a source of novel compounds with sufficient antinociceptive/anti-inflammatory effects [1]. Due to their characteristics, such as diminished side effects and reduced chances of causing addiction, natural products are promising therapeutic agents because of their stunning pharmacological features [2].

A rich natural resource of active compounds is the crude venom of the genus *Conus* [3]. The venom of each species of this enormous genus contains a mixture of unique short diversified bioactive peptides, figuratively called conopeptides [4]. Cone snails use them to immobilize prey and predators by targeting their cellular receptors [5]. According to their favorite prey type, they are categorized into three categories, namely piscivorous snails (fish hunters), molluscivorous snails (mollusk hunters) and vermivorous snails (worm hunters) [6]. 

Conopeptides can highly selectively target voltage-, ligand-gated ion channels and G-protein-coupled receptors in the nervous system of envenomated animals, causing their inactivation or hyper-activation [7, 8]. Although there are approximately 50,000 conopeptides present in the venom of genus *Conus*, only 0.1 % of them are functionary recognized [9]. The target specificity of conopeptides was very helpful to discover and purify some bioactive conopeptides that were developed as therapeutic drugs directed against many human disorders, as intractable pain, ischemic brain damage, migraine and some forms of epilepsy [10]. The first polypeptide component of cone snail venom with pharmacological evidence was identified and purified by Spence et al. [11]. One of the most effective conopeptides is ω-MVIIA isolated from *C. magus*, which targets calcium channels and was approved in 2004 as a drug under the commercial name Prialt® [6, 12]. Many recent researchers and pharmacologists have started to focus on vermivorous *Conus* species besides piscivorous and molluscivorous due to their low toxicity on humans, giving them highly promising safe opportunities, especially in the field of human pharmacology [13]. 

The main goals of this study were to assess the pharmacological activity (analgesic, anti-inflammatory and antipyretic) and cytotoxic assays (oxidative stress biomarkers and antioxidants concentrations) of different doses of *C. virgo* crude venom on different experimental animals. Additionally, the resulting data may enable us to understand the mechanisms of action of *C. virgo* crude venom. Moreover, these data provide a baseline reference for the medical community to get a potential therapeutic benefit of this venom.

## Methods

### Venom preparation

Live specimens (n = 45) of *Conus virgo* were collected using a trawl net from a depth of 1-2.5 m from different sites of Marsa Alam, Red Sea, Egypt, taking into account ethical local guiding principles. Snail venom glands (venomous gland, venom duct, bulb and proboscis) were dissected as described by Cruz et al. [14], and the crude extract was suspended in 2 mL of 0.05% (v/v) trifluoroacetic acid (TFA; Sigma-Aldrich, Poole, UK) and then centrifuged (15000 g, 10 min, 4 °C). The pellet was rewashed three times with 2 mL of 0.05% TFA, and the final supernatants were pooled, filtered through 0.22-µm filter membranes (Millipore, Watford, UK) and finally lyophilized and stored at -20 °C. 

### Animals and ethical approval

Swiss-Webster adult male albino mice weighing 20 to 25 g and 120 to 140 g adult male albino rats were used in this study. They were maintained in polyethylene cages under controlled temperature and humidity (22 ± 2 °C) and on a 12 h-light/dark cycle, with free access to standard laboratory chow and water. All procedures of animal care and maintenance followed international guiding principles for Animal Research and were supervised by the Bioethics and Animal Ethics Committee, Suez Canal University (approval no. 2018032).

### Hemolytic activity and estimation of median lethal concentration (LC_50_)

For erythrocytes suspension, 5 mL of EDTA freshly drowned human blood was centrifuged for five minutes at 14000 rpm. Plasma was discarded, and 5 mL of saline solution (150 mm NaCl) were added to the precipitated RBCS and applied to 2000 rpm centrifugation for ten minutes. Then, the supernatant was discarded, and the process was repeated three times [15]. One hundred microliters of six ascending concentration series of *C. virgo*crude venom were added to the prepared erythrocyte suspension solution Samples (450 μL erythrocytes/450 μL saline solution) and incubated at 37 °C for 60 min. The negative control tube contained 50% erythrocytes suspension and saline (v/v), and the positive control one contained 50% erythrocytes suspension and saline (v/v) in addition to an equal volume containing 50% of the non-ionic detergent, Triton X-100 (Sigma-Aldrich). The optical density of lysed red cells was measured spectrophotometerly at 550 nm. The hemolysis percent was calculated using the equation of Almaaytah et al. [16].

### Pharmacological assays

Fractions of LC_50_ of *C. virgo* crude venom (1/5 and 1/10 LC_50_, 9.14 and 4.57 mg/kg body weight respectively) were injected intraperitoneally into the experimental animals to evaluate its pharmacological activities, comparing with a control group that received 0.9% physiological saline, and a standard drug group that received suitable dose of a reference drug. Pharmacological activities were assessed at different time intervals with four assays, analgesic (central and peripheral), anti-inflammatory and antipyretic assays. 

*Central analgesic assay (tail immersion test)*

Four groups of mice (n = 6) were divided into a negative control group, groups treated with venom doses, and a group that received standard drug (nalbuphine, 1 mg/kg). The lower 3 cm portion of the tail of each mouse was immersed in a hot water beaker kept at 50 ± 0.5 °C, and the time of tail withdrawal (or reaction time) was recorded in seconds, at (0 min) and after 15, 30, 45, 60 and 75 min of the administration. The maximum time of each immersion did not exceed 15 seconds to prevent thermal injury to animals [1^7^].

*Peripheral analgesic assay (acetic acid-induced writhing test)*

A writhing movement is a tension of the abdominal muscles (peripheral muscles) in addition to the hind limb extension [18]. Experimentally, according to Koster et al. [19], writhing is induced by intraperitoneal injection of glacial acetic acid to assess the peripheral analgesic effect of a pre-treated drug. Twenty-four albino mice were divided randomly into 4 groups (n = 6) as in the previous assay, and the standard drug group received ketoprofen (0.5 mg/kg IP) as a reference. After 30 minutes, all mice were intraperitoneally injected with 0.6% acetic acid solution (10 mL/kg, Nasr Pharm Company, Egypt), then the number of writhing and abdominal stretching of mice were recorded over 30 minutes for each animal. Inhibition of writhing refers to the analgesic effect, and it was calculated according to Koster et al. [19] equation.

*Anti-inflammatory assay (paw edema assay)*

The anti-inflammatory activity of *C. virgo* crude venom was evaluated using the carrageenan-induced edema method described by Sheth et al. [20]. One percent carrageenan was prepared one hour before the experiment by dissolving 50 mg of carrageenan powder in 5 mL of 0.9% NaCl. 30 albino mice were divided randomly into 5 groups (n = 6). A negative control group received saline, and the positive control group received saline before being injected with carrageenan latterly. The treated groups received 1/10, 1/5 LC_50_ of venom, and the standard drug group received ketoprofen (0.5 mg/kg) as a reference drug. The paw thickness was measured for each mouse at zero time. After one hour, the plantar surface of the right hind paw of each mouse in treated groups was injected subcutaneously with 50 μL of 1% prepared carrageenan. Paw thickness was recorded at 1, 2, 3 and 4 hours after carrageenan administration, using skin digital caliper. The anti-inflammatory activity was calculated as percent of inhibition of paw edema using the ratio of Girard et al. [21].

*Antipyretic assay (yeast-induced pyrexia test)*

According to the model of Loux et al. [22] for evaluating antipyretic activity, 24 male rats were randomly divided into four groups (n = 6), and rectal control temperature was recorded, then received subcutaneously 20% aqueous suspension of dried Brewer’s yeast (*Saccaromyces cerevisiae*) in 0.9% saline in the back below the nape of the rat neck, with massage in the injection site to spread the suspension beneath the skin. Food was forbidden during the experiment time (18 hours), to ensure the induction of yeast fever. Then, rats were injected intraperitoneally as follows: the control group received saline, the treated groups received 1/10, 1/5 LC_50_ of the venom and the standard drug group received ketoprofen (0.5 mg/kg). Rectal temperature was measure after 0, 1, 2, 3 and 4 hours of treatment using a digital thermometer. The percentage reduction in rectal temperature were calculated.

### Cytotoxic assays

Adult albino mice were injected intraperitoneally with crude *C. virgo* venom (1/2 LC_50_, 22.85 mg/kg BW, n = 6), and a range of biochemical assays was applied to determine the cytotoxic effects of venom after 4, 8, 12, and 24 hours of administration, compared with the control group (0.9% saline). These assays were oxidative stress biomarker assays [plasma lipid peroxide (LP) and liver nitric oxide (NO) levels], and antioxidant assays [reduced glutathione (GSH) contents, glutathione peroxidase (GPx) and catalase (CAT) activities in blood]. 

*Lipid peroxidation assay*

Plasma LP was detected by Yagi [23] method by following thiobarbituric acid (Winlab, UK) reaction with the lipid fractions resulted of peroxidising damage of cell membrane lipids (e.g. malonyldialdehyde, MDA) and measuring the absorbance of lipid fractions at 532 nm. The results were expressed as µmol of MDA/mL plasma. An external standard of malonaldehyde bis (dimethyl acetal, Sigma) was used in the assay.

*Nitric oxide assay*

Nitric oxide (NO) was evaluated using the Griess reaction [24]. One gram of liver tissue was homogenized in 1 mL of potassium phosphate buffer (100 mM potassium phosphate/2 mM EDTA, pH 7.0), centrifuged at 4000 g for 15 min at 4 °C. The supernatant was mixed with V/V of Griess reagent (1 part 0.1% naphthylethylendiamine dihydrochloride in dist. water added to 1 part 1% sulfanilamide in 5% H_3_PO_4_), for 10 min at room temperature. The absorbance was measured at 540 nm. Sodium nitrite (50 µmol/L) was used as a standard.

*Reduced glutathione assay*

The blood content of reduced glutathione (GSH) was estimated according to Beutler et al. [25] method. About 0.2 mL of freshly drowned blood was added to 1.8 mL distilled water and 3 mL of precipitating solution (1.67 g glacial metaphosphoric acid, 0.2 g EDTA and 30 g NaCl in 100 mL distilled water). The mixture was centrifuged (2200 g, 15 min, 4 °C). Then, 1 mL of supernatant was added to 4 mL of Na_2_HPO_4_ (0.3 M) and 0.5 mL of dithiobis-2-nitrobenzoic acid reagent (DTNB, Sigma-Aldrich) (40 mg DTNB/100 mL 1% sodium citrate) and the absorbance was measured at 405 nm. Glutathione reduced (Sigma-Aldrich) was used as an external standard.

*Glutathione peroxidase assay*

Glutathione peroxidase (GPx) activity was measured in blood using the method of Paglia and Valentine [26]. Generally, GPx reacts with organic intracellular peroxide compounds (as hydroperoxides) and neutralizes them, and turned into oxidized glutathione (GSSG), which is recycled to reduced form again by the action of glutathione reductase enzyme in the presence of NADPH, which oxidized to NADP^+^, and causes decreased absorbance when measured at 340 nm. Glutathione peroxidase (0.5 U) was used as an external standard.

*Catalase assay*

 Catalase enzyme is found in most of aerobic cells to scavenge hydrogen peroxide, which is a potent reactive mediator and causes cellular damage [27]. Catalase activity was assayed according to the method of Aebi [28], in which catalase/H_2_O_2_ reaction time is limited to one minute only. The residue of H_2_O_2_ that wasn’t depleted during the reaction interacts with 3,5-dichloro-2-hydroxybenzene sulfonic acid (DHBS) in the presence of 4-aminophenazone and with the catalyst of peroxidase to form chromophore, which its color density reversely proportional with the concentration of catalase in the sample, and detected at 240 nm.

### Statistical analysis

SPSS® statistical software (v. 20.01 SPSS Inc., Illinois, USA) was used in all data analyses [29]. Differences in the effects of *Conus* venom between control and treated groups of mice were assessed using the Student's unpaired t-test [30]. The probability criterion for significance for each statistical test was (*p* ≤ 0.05).

## Results

**Figure 1.**
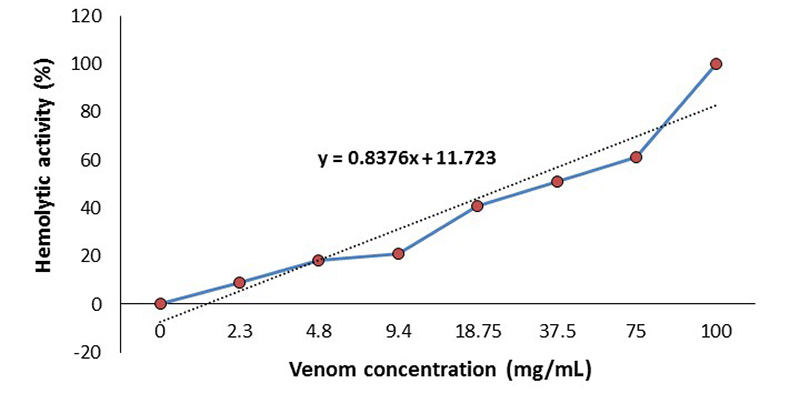
Diagram of the hemolytic activity of *Conus virgo* venom in order to calculate the mean hemolytic concentration (LC_50_ = 45.7 mg/mL).

The approximate mean hemolytic concentration of *C. virgo* crude venom was calculated to be 45.7 mg/mL (Figure 1). One fifth and 1/10 of LC_50_ (9.14 and 4.57 mg/kg respectively) have been used in the pharmacological assays of this venom, and 1/2 of LC_50_ (22.85 mg/kg) has been used in the cytotoxic assays. The low doses were chosen to avoid cellular damage and resemble the purposed effects of therapeutic drugs, while the high dose was chosen to exhibit the cellular damage that possibly results from the venom treatment. All figures were created using licensed Microsoft Office Excel (v. 2010).

### Pharmacological effects of *C. virgo* crude venom

**Figure 2.**
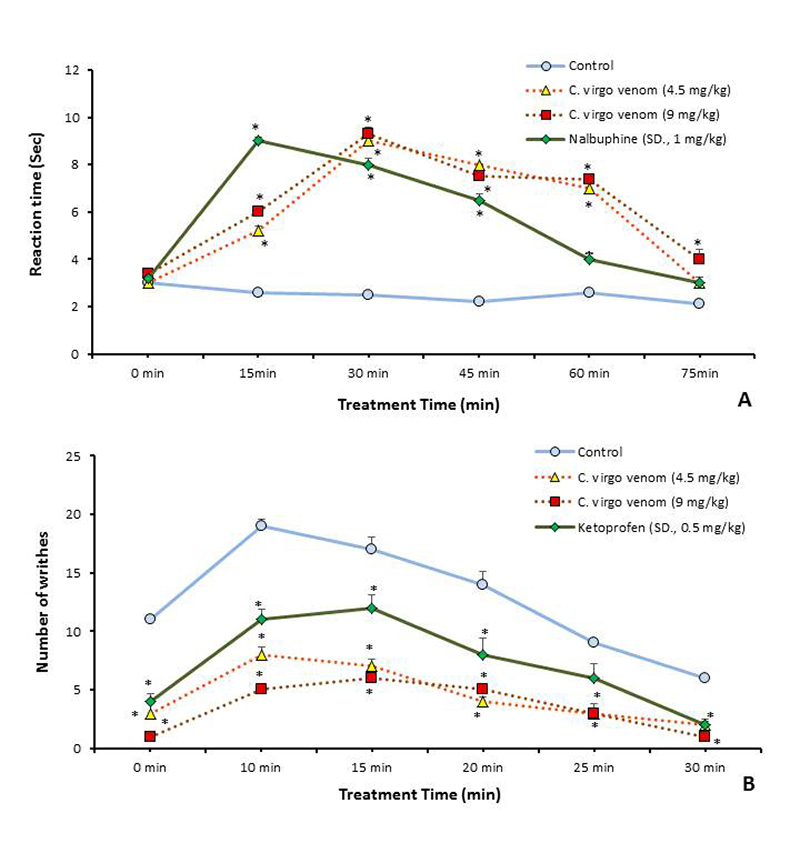
Analgesic effects of *C. virgo* venom on mice - using two doses (9.1 and 4.5 mg/kg) and standard drugs - compared with control. **(A)** Central analgesic effect using tail immersion test. **(B)** Peripheral analgesic effect using acetic acid-induced writhing test. Data are represented as mean ± SE (n = 6). *Significant difference between normal control and treated groups, using unpaired Student t-test (*p* ≤ 0.05).

The analgesic effects (central and peripheral) of both doses of *C. virgo* venom (9.17 and 4.58 mg/kg) are presented in Figures 2A and 2B, respectively.

Results of Figure 2A indicate that treated groups with both doses of *C. virgo* (1/10 and 1/5 LC_50_) showed a significant increase (*p* ≤ 0 .05) in time latency in comparison with the control group (for 15, 30, 45 and 60 min time intervals), and the maximum antinociceptive effect (highest percent of change, + 262.9 and + 275.0%, respectively) occurred after 30 min of venom treatment. The venom treated mice with both doses showed a significantly higher analgesic effect than those treated with the standard drug nalbuphine after 60 min of treatment. Generally, venom treatment caused a slower but more potent analgesic effect and long-lasting than the standard drug in the tail immersion test. Moreover, the results showed no significant difference between treatment with low and high venom doses in this assay.

In addition, the peripheral analgesic effect shown in Figure 2B - and examined by counting the number of abdominal writhing movements induced with acetic acid intraperitoneal injection in mice - indicated that the venom treatments for both doses caused a significant decrease (*p* ≤ 0.05) in the writhing movements compared with the control group, in all-time intervals. The low venom dose exhibited a more significant writhing reduction (*p* ≤ 0.05) than the standard drug ketoprofen after 15 and 20 min of treatment. In contrast, the high venom dose showed a significant effect of writhing reduction (*p* ≤ 0.05) than the standard drug at 30 min. and all later time intervals. The high venom dose showed a significant potent effect (*p* ≤ 0.05) more than the low dose after 10 minutes.

**Figure 3.**
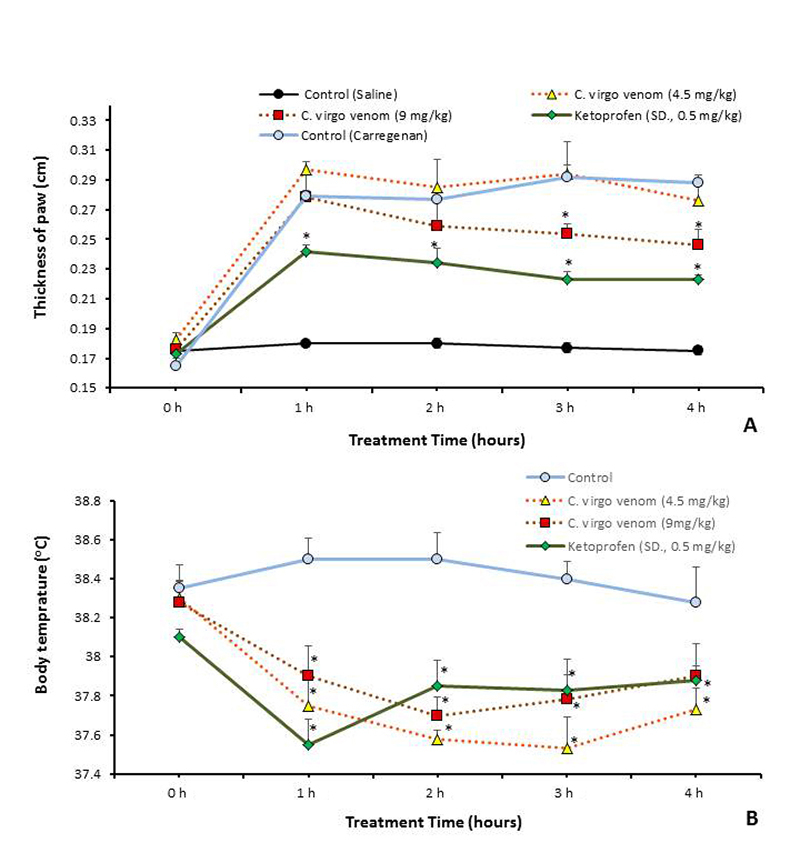
Pharmacological activities of *C. virgo* venom - using two doses (9.1 and 4.5 mg/kg) and standard drugs - compared with control. **(A)** Anti-inflammatory effect using paw edema test on mice. **(B)** Antipyretic effect using yeast-induced pyrexia test on rats. Data are represented as mean ± SE (n = 6). *Significant difference between normal control and treated groups, using unpaired Student t-test (*p* ≤ 0.05).

Using measurement of edema in mice paw induced with carrageenan injection, and examining the anti-inflammatory effect of treatment with *C. virgo* doses are displayed in Figure 3A. Only the high venom dose showed significant inhibition of edema (*p* ≤ 0.05) after 3 and 4 hours post-treatments, compared with the carrageenan control group, while the low dose effect is negligible (maximum + 6.4%). Administration of the standard drug ketoprofen showed a more potent edema reduction effect than treatment with both venom doses.

After 18 hours of brewer’s yeast intraperitoneal injection in rats, mean rectal temperature was higher than 38 °C. Treatment with *C. virgo* venom and standard drugs are demonstrated in Figure 3B. Both venom doses caused a significant decrease in pyrexia (*p* ≤ 0.05) after all-time intervals in comparison with the carrageenan-control temperatures. There was no significant difference between treatment with venom doses and the standard drug ketoprofen. The low venom dose was significantly more efficient than the high dose (*p* ≤ 0.05) in reducing the rat’s elevated rectal temperature. 

### Cytotoxic effects of *C. virgo* crude venom

**Figure 4.**
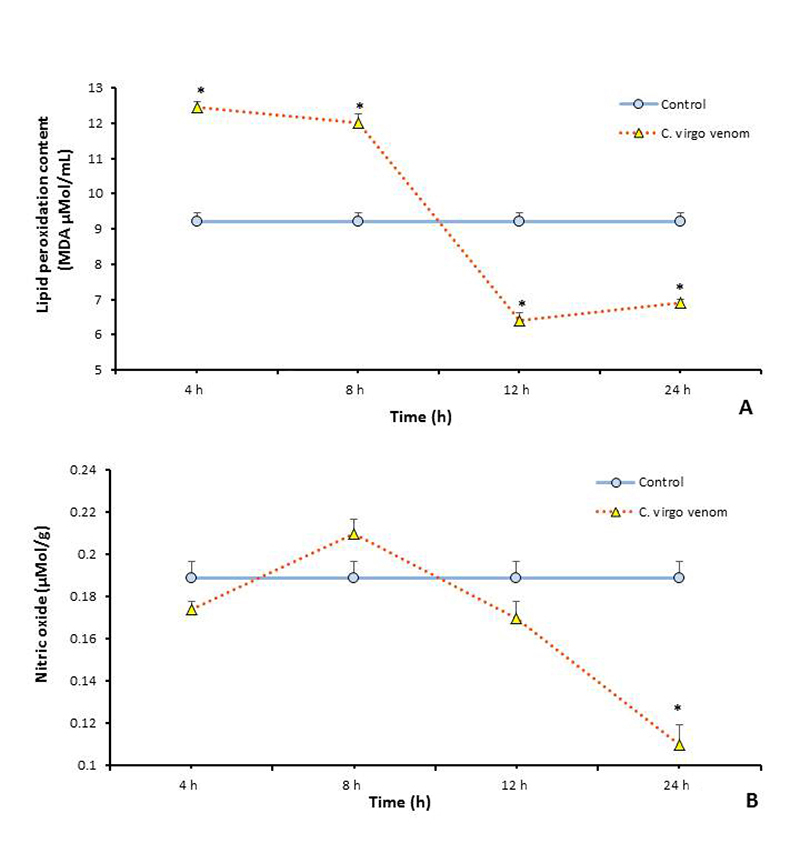
*C. virgo* venom (22.85 mg/kg) induced intracellular oxidative stress in mice assessed by **(A)** lipid peroxidation level in plasma, **(B)** nitric oxide level in liver. Data are represented as mean ± SE (n = 6). *Significant difference between normal control and treated groups, using unpaired Student t-test (*p* ≤ 0.05).

There are several cytotoxic assays of oxidative stress can be chosen to detect the cellular toxicity caused by *C. virgo* venom (22.85 mg/kg) treatment. Lipid peroxidation (LP) and nitric oxide (NO) concentrations were selected as biomarkers of this cytotoxicity, and their results are illustrated in Figure 4A and 4B, respectively. 

It was observed that after venom treatment, plasma lipid peroxide concentration (measured by MDA µmol/mL) significantly increased (*p* ≤ 0.05) after 4 and 8 hours and then reduced significantly after 12 and 24 hours. The highest effect of venom administration was after 4 hours (+35% of change), compared with the control level. On the other hand, nitric oxide did not show any significant change (*p* ≤ 0.05) after venom treatment except after 24 hours, with percent equals (-41%), compared with the control group. 

**Figure 5.**
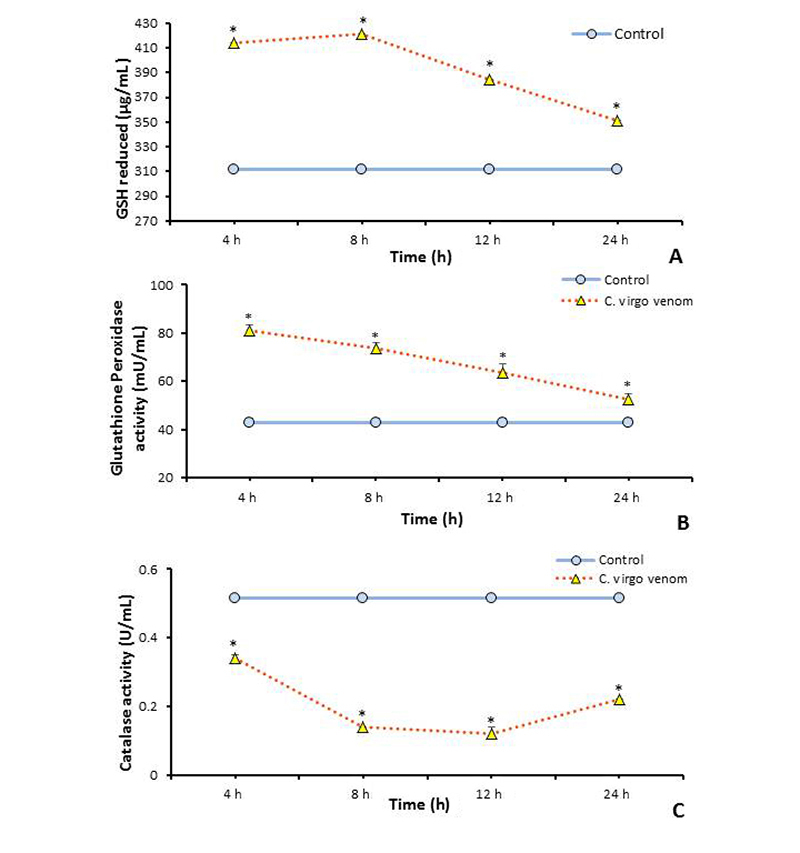
Change in the measurements of antioxidants after administration of *C. virgo* venom (22.85 mg/kg). **(A)** Reduced glutathione content in blood. **(B)** Glutathione peroxidase activity in blood. **(C)** Catalase activity in blood. Data are represented as mean ± SE (n = 6). *Significant difference between normal control and treated groups, using unpaired Student t-test (*p* ≤ 0.05).

The *in vivo* effect of *C. virgo* venom administration on the concentrations and activities of cellular oxidative defense components (GSH, GPx and CAT) was examined and illustrated in Figures 5A, 5B and 5C, respectively. The level of blood GSH was significantly elevated (*p* ≤ 0.05) at all-time intervals post venom administration, compared with control group, and reached maximum value after 8 hours (+35%) of venom injection. Also, the activity of GPx was significantly (*p* ≤ 0.05) increased at all-time intervals of venom treatment, compared with control values, with the maximum activity (+88.5%) at the first time interval. While there was a significant diminishing in the activity of CAT at all-time intervals and reached -77% of the control value after 12 hours.

## Discussion

Mollusks of the genus *Conus* are predatory marine snails that cause paralysis to worms, other mollusks or fish prey by owning specialized diversified venom. Single crude venom may be composed of 50-200 unique short peptides (10-80 amino acids), called figuratively conopeptides [31]. These conopeptides are distinctive by containing disulfide bonds between cysteine (Cys) residues. According to the richness of these bonds, conopeptides are classified into disulfide-rich conotoxins and disulfide-poor conopeptides as conopressins and contryphans [32, 33]. They target a wide range of cellular receptor types of the envenomated animal [5], as voltage- and ligand-gated ion channels and G-protein-coupled receptors in their nervous system [7]. Despite the harmful effect of conopeptides, their therapeutic effects are being now investigated in many cases like pain, Alzheimer’s, and Parkinson’s disease, as well as cardiac infarction, hypertension, and various neurological diseases [34, 35]. 

In the present study, *Conus virgo* was collected from Red Sea, Marsa Allam, Egypt. It was chosen as a new vermivorous venomous animal model, not much experimentally tested before, in order to assess the potential pharmacological activities and cytotoxic mechanisms of its crude venom, and to provide adequate medical baseline reference to discover novel drug with sufficient effect on pain and inflammation from its venom. The crude venom toxicity and lethality were examined by detecting LC_50_ on the human blood, which was found to be higher than many other *Conus* species, like *Conus flavidus* [36] and *Conus betulinus* [37]. This assumed a high safety profile of *C. virgo* rather than many other *Conus* species. 

Generally, pain is an uncomfortable sensation, although it could be a defensive mechanism generated as a result of synthesizing of some mediators like cyclooxygenases and prostaglandins [38]. Pharmacists are facing a huge challenge in meeting the progressive medical needs of an adequate pain-controlling drug. Many synthesized small molecules failed to reach the required levels of selectivity and potency. So, drug discovery has returned to the natural products to replenish drugs with sufficient analgesic effect [1]. Although humanity has well-known vilified experiences of pain inflected by the stings of venomous animals, their venoms contain many peptides with immense therapeutic potential. These peptides are the most relevant pain therapeutics because they target specific classes of protein receptors on the human neuron cell membranes [39]. 

Among the numerous numbers of conopeptides, there is a family called (ω-conotoxins) contains peptides composed of a few amino acids (24-34) and have high affinity and selectivity for different voltage-gated Ca^2+^ channels (VGCCs) [40]. MVIIC and MVIID ω-conotoxins isolated from *C. magus* could preferentially block the N- and P/Q- types of VGCCs [41]. Considering the high selectivity of ω-conotoxins to blocking a specific type of VGCCs, many experimental attempts were performed to discover their potential therapeutic abilities on and some pharmaceutical companies began in development them as analgesic drugs [42]. Recently, venoms of many cone snail species have evidenced their analgesic efficacy, as *Conus moncuri* [43] *Conus striatushad* [44].

The analgesic effect of *Conus virgo* crude venom was studied on central and peripheral nervous reflexes. The evaluation of central analgesic effect was studied by the tail-immersion test, which detects the activity of the nociceptive receptors in spinal reflex induced by acute thermal stimulants [45]. The prolonged time of tail immersion after venom treatment indicates its significant central analgesic effect. The peripheral analgesic effect was evaluated by counting writhing contractions after intraperitoneal injection of acetic acid [46]. The significant reduction of writhing movements followed *C. virgo* venom treatment reflects its peripheral analgesic ability. The acid-sensitive ion channels (ASICs) of peritoneal mast cells and prostaglandin secreting cells provoke a writhing movement after acetic acid injection [47]. These H^+^-gated Na^+^channels are generally considered principal players in the pain pathway [48]. Using various venoms as a starting source to identify new modulators of ASICs, Diochot et al. [49] discovered two peptides in the venom of the Black Mamba snake that caused a potent and reversible blockade of ASIC1a. 

In Table 1, all conopeptides previously isolated from *C. virgo* are listed with their sequences. The molecular functions of some of them were well described, such as κ-ViTX, that inhibit voltage-gated potassium ion channels (VGKCs) [50], α-ViIA blocks nicotinic Acetyl Choline receptors [51] and Contryphan-Vi impairs VGCCs and calcium-dependent potassium channels (KCa) [52]. The molecular functions of other *C. virgo* conopeptides are not well investigated and established [53-56], but some of them can predict their function according to their Cys-motif framework and superfamilies, such as ViKr92 [57], conotoxin 3 and conotoxin 10 [58]. These conotoxins belong to O1-superfamily, which well firmly known to contain four types of conotoxins [30]; δ-conotoxins (delayed inactivation of Voltage-gated sodium ion channels, VGSCs), μo-conotoxins (inhibition VGSCs) [59], κ-conotoxins (block VGKCs) [60] and ω-conotoxins (block CaCs) [61]. One of these O1-superfamily conotoxins could be the cause of peripheral analgesic activity of *C. virgo* venom, as it could target an isoform of ASICs in the mouse abdomen, causing a reduction in prostaglandin secretion and subsequently reducing writhing movements. Also, the presence of Contryphan-Vi alongside the O1-superfamily’s conotoxins could infer the central analgesic effect of *C. virgo* venom by targeting CaCs in the mouse’s nervous system. Further analysis and peptide purification are required to detect which peptides are responsible of these effects. 

**Table 1.** List of previously described conopeptides derived from *Conus virgo* venom, including their sequences, calculated length (aa) and monoisotopic masses (Da), cysteine frameworks, *Conotoxin* superfamilies and molecular functions.

Name (UniProtKB)	Sequence	Length (aa) and mol. weight (Da)	Cys. framework	Conotoxin superfamily and family	Molecular function	Reference
**Kappa-ViTX**	MMFRLTSVSCFLLVIACLNLFQVVLTSRCFPPGIYCTPYLPCCWGICCGTCRNVCHLRIGKRATFQE	67 7,599	XI (C-C-CC-CC-C-C)	(I2) S.Family κ-Conotoxin	Impairing of VG-K^+^ ion channels	[50]
**Alpha-ViIA**	MGMRMMFVVFLLVVFASSVTLDRASYGRYASPVDRASALIAQAILRDCCSNPPCAHNNPDCR	62 6,810	I (CC-C-C)	(A) S.Family α-Conotoxin	Inhibiting nAChR	[51]
**Contryphan-Vi**	MGKLTILVLVAAVLLSTQVMVQGDGDQPADRNAVRRDDNPGGLSGKFMNVLRRSGCPWHPWCG	63 6,806	C-C	(O2) S.Family Contryphan Family	Impairing VG-Ca^2+^ and KCa channels	[52">]
**ViVA**	MRCVPVFIILLLLIPSASSAAVQPKTEKDDVPLASVHDSALRILSRQCCITIPECCRIG	59 6,393	V (CC-CC)	(T) S.Family	Unknown	[53]
**ViVB**	MRCVPVFIILLLLIPSAPSAAVQPKTEKDDVPLASFHDSAMRILSRQCCPTIPECCRVG	59 6,439	V (CC-CC)	(T) S.Family	Unknown	[53]
**Vi1361**	QCCPTMPECCRI	12 1,384	V (CC-CC)	(T) S.Family	Unknown	[54]
**Vi5.1a**	MRCVPVFIILLLLIPSAPSADAQPKTKDDVPLASYHDNAERTLQRLWNQRHCCPIDLPCCPPG	63 7,039	V (CC-CC)	(T) S.Family	Unknown	[55]
**Vi5.1b**	MLCVPVFIILFIIIPFAPTSESQPKTKEEVAKASVHDNAERTLQRLWNQSHCCPIDLQCCPPG	63 7,065	V (CC-CC)	(T) S.Family	Unknown	[55]
**Vi15a**	MMPVILLLLLSLAIRCADGKAVQGDSDPSASLLTGDKNHDLPVKRDCTTCAGEECCGRCTCPWGDNCSCIEWGK	74 7,887	XV (C-C-CC-C-C-C-C)	(V) S.Family	Unknown	[56]
**ViKr92**	MKLTWMMIVAVLFLTAWTFVTADDTRYKLENPFLKARNELQKLEASQLNERGCLDPGYFCGTPFLGAYCCGGICLIVCIET	81 9,148	VI/VII (C-C-CC-C-C)	(O1) S.Family δ-, μo-, κ- or ω-conotoxins	Targets different types of ion channels	[57]
**Conotoxin 10**	MKLTCVLIITVLFLTASQLITADYSRDQRQYRAVRLGDEMRNFKGARDCGGQGEGCYTQPCCPGLRCRGGGTGGGACQL	79 8,508	VI/VII (C-C-CC-C-C)	(O1) S.Family δ-, μo-, κ- or ω-conotoxins	Targets different types of ion channels	[58]
**Conotoxin 3**	MKLTCVLIITVLFLTASQLITADYSRDQRQYRAVRLGDEMRNFKGARDCGGQGEGCYTQPCCPGLRCRGGGTGGGVCQL	79 8,536	VI/VII (C-C-CC-C-C)	(O1) S.Family δ-, μo-, κ- or ω-conotoxins	Targets different types of ion channels	[58]

Inflammation is one of the regular host responses that occur in living tissues when exposed to pathogenic factor, wound, toxicity or any infectious agent [62]. Blood flow increases at the inflamed site to facilitate transportation of necessary white blood cells, antibodies, cytokines, and complements required to treat injury, causing edema as a prime sign of inflammation [63]. It has been documented that carrageenan-induced mice paw edema is a suitable *in vivo* model to predict the value of anti-inflammatory agents, which act by inhibiting the mediators of acute inflammation. This method has frequently been used to assess the anti-edematous effect of natural products [64]. Two phases have been reported during this inflammation. The early phase occurs by releasing histamine, serotonin and kinin in the first hour of inflammation, and the late phase (2-4 hrs) has been reported to be a result of overproduction of prostaglandins, bradykinin and lysosome-like substances [65]. The result of current study established that only high concentrations of *C. virgo* crude venom have a mild anti-inflammatory effect at the late stage of inflammation. This can be interpreted as this venom could inhibit secretion of prostaglandin, which supports the previous analgesic results. Some cone snail species showed similar anti-inflammatory effects, as *Conus magus* [66] and *Conus vexillum* [67]. Different types of venom also had alleviation effect of inflammation like *Pardosa astrigera* spider venom [68] and venom of *Hydrophis cyano* [69].

Pyrexia or fever is an elevation of body temperature than the normal range, and it is a protective response that may result from trauma, infection and wound, which leads to activate inflammatory mediators that cause the synthesis of prostaglandins, which stimulate the hypothalamus to raise body temperature [70]. The inhibition of prostaglandin effect can be achieved efficiently by blocking the cyclooxygenase enzyme activity [38]. The current study showed that, after 4 hours of venom treatment the rat’s body temperature significantly decreased affirmed the previous suggestion of the *C. virgo* venom ability to decrease prostaglandin effects.

On the other hand, the mechanism of tissue damage induced by *Conus* venom remains unclear. Cerebral edema, liver damage, hemorrhage and vascular congestion in kidneys and lungs, and inhibition/activation of certain cellular enzymes have been reported [71] after *Conus loroisii* envenomation. However, there is no evidence binding these effects with the direct cytotoxicity in these organs. So, we hypothesized that *Conus* toxins could induce the generation of free radicals responsible for cellular membrane damage in treated organs. 

In order to test the above hypothesis, oxidative damage was evaluated in terms of its biomarkers as lipid peroxidation and nitric oxide. They were decreased significantly in the venom treated animals. Similar results were obtained after treatment with *Conus vexillum* [72] and *Conus flavidus* [36]. It could be an indication of excessive production of reaction oxygen species (ROS) followed by oxidative damage in *Conus* envenomed animals. 

The excessive production of ROS can disturb the cellular scavenging system of endogenous antioxidant compounds, leading to oxidative stress signs [73]. In the present study, glutathione (GSH) level, glutathione peroxidase (GPx) and catalase activities were measured in the treated animals’ blood, as the primary antioxidants help the body in ROS scavenging. Glutathione is the most abundant intracellular thiol-based non-enzymatic antioxidant [74], while GPx is a selenium-containing antioxidant enzyme which scavenges the free radical by making two-electron reduction of hydroperoxides. GPx catalyzes the reduction of hydrogen peroxide to water and oxygen and catalyzes the reduction of peroxide radical to alcohol and oxygen [75]. Also, catalase enzyme is one of the most crucial enzymes involved in the enzymatic anti-oxidant defense system of aerobic cells by scavenging hydrogen peroxide, which is a potent reactive mediator and causes cellular damage [27].

The present work assumed that, the depletion of catalase activity is a clear indication of the potent impact of ROS generated after venom treatment. This is similar to the study of Kebir-Chelghoum and Laraba-Djebari [27] of *Cerastes cerastes* snake venom, Abdel-Rahman et al. [72] of *Conus vexillum* and Salman and Hammad [76] of *Leiurus quinquestriatus* scorpion venom. Whereas the remarkable elevation in the levels of GSH and GPx in treated animals was probably a necessary defensive response to encounter deleterious effects of these ROS.

Oxidative damage of *Conus* venom could be associated with its active ingredient phospholipase A2 (PLA2). McIntosh et al. [77] isolated phospholipase A2 (conodipine-M) from the venom of *C. magus*. Conodipine-M displayed properties similar to those of previously characterized PLA2 from snake venom. Phospholipids hydrolysis by PLA2 enzyme releases arachidonic acid, whose metabolism results in potentially toxic ROS and lipid peroxides formation [78]. Ayala et al. [79] have reported that the increased polyunsaturated fatty acids liberated from the venom-treated tissues may subsequently increase the peroxidation rate of lipid, which might be responsible for tissue damage

## Conclusion

Taken together, the results of the current study indicate that the crude venom of *Conus virgo* could contain bioactive components that have considerable pharmacological activities, especially analgesic and antipyretic. Such effects are due to the inhibition of prostaglandin secretion, with low and tolerable cytotoxic side effects. Accordingly, these bioactive components may have potential medical benefits and should be taken into account to be purified and identified to be developed as therapeutic drugs that would control pain and pyrexia.
